# Bacteriophages Infecting Gram-Negative Bacteria in a Commercial Cucumber Fermentation

**DOI:** 10.3389/fmicb.2020.01306

**Published:** 2020-06-26

**Authors:** Zhongjing Lu, Ilenys M. Pérez-Díaz, Janet S. Hayes, Fred Breidt

**Affiliations:** ^1^Department of Molecular & Cellular Biology, Kennesaw State University, Kennesaw, GA, United States; ^2^United States Department of Agriculture, Agricultural Research Service, Washington, DC, United States; ^3^Department of Food, Bioprocessing & Nutrition Sciences, North Carolina State University, Raleigh, NC, United States

**Keywords:** bacteriophage, Gram-negative bacteria, *Enterobacteriaceae*, *Enterobacter*, cucumber fermentation

## Abstract

Cucumber fermentations are one of the most important vegetable fermentations in the United States. The fermentation is usually driven by lactic acid bacteria (LAB) indigenous to fresh cucumbers. But LAB are greatly outnumbered by many Gram-negative bacteria on fresh cucumbers, which may influence the growth of LAB and the incidence of bloater defect (hollow cavities formed inside fermented cucumbers) leading to serious economic loss to the pickle industry. Rapid elimination of Gram-negative bacteria is crucial to the dominance of LAB and the reduction of bloater defect in the fermentation. Various factors can affect the viability of Gram-negative bacteria in cucumber fermentation. Bacteriophages (phages) may be one of such factors. This study explored the abundance, diversity, and functional role of phages infecting Gram-negative bacteria in a commercial cucumber fermentation. Cover brine samples were taken from a commercial fermentation tank over a 30-day period. On day 1 and day 3 of the fermentation, 39 Gram-negative bacteria and 26 independent phages were isolated. Nearly 67% of Gram-negative bacterial isolates were susceptible to phage infection. Phage hosts include *Enterobacter*, *Citrobacter*, *Escherichia*, *Pantoea*, *Serratia*, *Leclercia*, *Providencia*, and *Pseudomonas* species. About 88% of the isolated phages infected the members in the family *Enterobacteriaceae* and 58% of phages infected *Enterobacter* species. Eight phages with unique host ranges were characterized. These phages belong to the *Myoviridae*, *Siphoviridae*, or *Podoviridae* family and showed distinct protein profiles and DNA fingerprints. The infectivity of a phage against *Enterobact*e*r cancerogenus* was evaluated in cucumber juice as a model system. The phage infection at the multiplicity of infection 1 or 100 resulted in a 5-log reduction in cell concentration within 3 h and rapidly eliminated its host. This study revealed the abundance and variety of phages infecting Gram-negative bacteria, particularly *Enterobacteriaceae*, in the commercial cucumber fermentation, suggesting that phages may play an important role in the elimination of Gram-negative bacteria, thereby facilitating the dominance of LAB and minimizing bloater defect. To our knowledge, this is the first report on the ecology of phages infecting Gram-negative bacteria in commercial cucumber fermentations.

## Introduction

Vegetable fermentation plays an important role in preserving foods and providing variety to the diet as well as enhancing the nutrient content of the food. Cucumber pickles are the most commonly consumed fermented vegetable in the United States and many other countries. Today, each American eats an average of 9 pounds of pickles a year (Kaplan, 2014). An average production of pickles in the United States is around 480,000 tons in 2012–2014 with an economic value between 145 and 175 million dollars (Anonymous, 2015). The global pickles market reached a value of US$10.3 Billion in 2018, and the market is projected to cross US$12.3 Billion by 2024, according to the latest report by the International Market Analysis Research and Consulting Group (Anonymous, 2019).

Most cucumber fermentations are driven by lactic acid bacteria (LAB) naturally present on fresh cucumbers. The metabolic activities of LAB determine the quality and safety of the final products (Breidt et al., 2013a). Although LAB are naturally associated with fresh cucumbers, their initial concentration is usually below 0.4% of the bacterial population (Pérez-Díaz et al., 2019) and can be as low as 0.01–0.1% of the total microbiota (Mundt et al., 1967; Mundt and Hammer, 1968; Mundt, 1970). A wide variety of other bacteria greatly outnumber LAB. Gram-negative bacteria are typically predominant on fresh cucumbers (Pederson and Albury, 1950; Mundt et al., 1967; Mundt and Hammer, 1968; Mundt, 1970; Pérez-Díaz et al., 2019). *Enterobacter* and many other members in the *Enterobacteriaceae* family, *Pseudomonas*, and *Providencia* are frequently isolated during the early stage of cucumber fermentation (Veldhuis and Etchells, 1939; Etchells, 1941; Samish et al., 1957, 1963; Pérez-Díaz et al., 2019). The dominance of Gram-negative bacteria can interfere with the growth of LAB resulting in slow fermentation and affect thequality and/or safety of fermented cucumbers. In addition, many Gram-negative bacteria such as *Enterobacter* can contribute to the formation of hollow cavities or gas pockets in the interior of whole fruits, a defect known as bloater. Bloater defect downgrades the product quality and results in serious economic loss to the pickle industry (Fleming, 1979; Zhai et al., 2018). Although pathogenic bacteria have not been reported in commercially fermented vegetable products, the survival of certain acid-resistant bacterial pathogens can occur (Breidt and Caldwell, 2011). Some Gram-negative bacteria such as *Salmonella* and *Escherichia coli* O157:H7 can cause foodborne illness. The key to ensure successful cucumber fermentation as well as other vegetable fermentations is to establish conditions to promote the growth of LAB over other microorganisms, particularly Gram-negative bacteria so that LAB can quickly dominate in the fermentation system before Gram-negative bacteria and other undesirable microorganisms have a chance to increase substantially in numbers and exert their effects.

It has long been thought that only certain physical and chemical factors (e.g., added salt, oxygen depletion, acids produced, and resulting low pH) affect the mortality of Gram-negative bacteria and other undesirable microbiota leading to the dominance of LAB in cucumber fermentations (Pederson and Albury, 1950; Pérez-Díaz et al., 2014). Biological factors such as phage infection are often overlooked. Phages are ubiquitous in nature and thus are a component of the microbiota in many habitats including fresh cucumbers and fermentation environments. Phages are natural killers of bacteria and can act as biological control agents. Therefore, phages can be an important factor regulating the abundance and distribution of bacterial populations, thereby influencing the microbial succession in cucumber fermentations. We previously investigated phages infecting LAB in commercial cucumber and sauerkraut fermentations (Lu et al., 2003b, 2012). We found abundant and diverse LAB phages present in those fermentations and explored their potential to influence the bacterial succession among LAB species. A recent study reported a phage isolated from cucumber fermentation infected a Gram-negative bacterium, *Escherichia coli* O157:H7 (Lu and Breidt, 2015). However, phages infecting other Gram-negative bacteria in cucumber fermentations have not been well studied. In this study, we explored the abundance, diversity, and ecological role of phages active against Gram-negative bacteria in a commercial cucumber fermentation, characterized eight phages isolated from the fermentation, and evaluated the infectivity of an isolated phage active against *Enterobact*e*r cancerogenus* (a frequently occurring bacterium) in cucumber juice as a model system. The data from this study may provide new insight into our understanding of the dynamic process in cucumber fermentations and the development of bloater control strategy in cucumber fermentations. To our knowledge, this is the first report of the ecology of phages infecting Gram-negative bacteria in a commercial cucumber fermentation.

## Materials and Methods

### Industrial Cucumber Fermentation and Sample Collection

A commercial cucumber fermentation tank (designated as Tank A, 40,000-L capacity) was examined in this study. The tank was packed with two sizes of cucumbers, size 3A (44–51 mm in diameter) and size 2B (32–38 mm in diameter). The recycled cover brine from previous cucumber fermentations was adjusted with 20% acetic acid and pickling salt in order to achieve 50 mM acetic acid and 2 M NaCl in the cover brine for Tank A. After equilibration between whole cucumbers and the cover brine, the concentrations of acetic acid and sodium chloride (NaCl) were 25 mM and 1.03 M (6%), respectively. The resulting pH was 4.4 at which the fermentation started. On each sampling day (days 1, 3, 7, 14, or 30), two cover brine samples were taken from two separate locations (0.61 and 2.44 m below the cover brine surface) in the fermentation tank. The samples were immediately transported to our laboratory on ice and processed on the same day.

### The Treatment of Cover Brine Samples for Host and Phage Isolations

One ml of each cover brine sample was saved for microbiological analysis and phage host isolation. An additional 50 ml of each cover brine sample was centrifuged at 13,000 × *g* (Eppendorf 5810R Centrifuge, Eppendorf North America, Inc., Westbury, NY, United States) and 4°C for 20 min to remove cells and solid particles. The pH of the supernatant was adjusted to approximately 6.3 with 5.0 N NaOH using a Fisher Accumet pH meter (model AR25, Thermo Fisher Scientific, Pittsburgh, PA, United States) equipped with a Gel-Filled Pencil-Thin pH Combination Electrode. The pH-adjusted supernatant was filtered brine through Nalgene filtration units with 0.45 μm pore size and temporarily stored at 4°C until used for phage isolation.

### Bacterial Concentrations During the Fermentation

Plate counts for presumptive *Enterobacteriaceae* and *Lactobacillus* were determined from Violet Bile Salt agar plates supplemented with 1% of glucose (VRBG) and de Man, Rogosa and Sharpe (MRS) agar plates supplemented with 1% of a 0.1% cycloheximide stock solution (Oxoid, Basingstoke, United Kingdom) as described by Pérez-Díaz et al. (2019). For comparison, the bacterial concentration on fresh cucumbers were also measured.

### Chemical Analyses

Lactic acid concentration and pH were measured as described by Pérez-Díaz et al. (2019). Briefly, lactic acid concentration was measured by high-performance liquid chromatography analysis using a 30-cm HPX-87H column (Bio-Rad Laboratories, Hercules, CA, United States) as described by McFeeters and Barish (2003). The column was heated to 37°C and eluted with 0.03N sulfuric acid at a flow rate of 1 ml/min. A Thermo Separations UV6000 diode array detector (Spectra System Thermo Scientific, Waltham, MA, United States) was used to collect data at 210 nm for the analysis of lactic acid. External standards were used to calibrate the system. The pH of each cover brine sample was measured using a Fisher Accumet pH meter (model AR25, Fisher Scientific).

### Isolation of Gram-Negative Bacteria and Their Phages

Colonies on VRBG agar plates were picked for purification, then stored and identified as described by Pérez-Díaz et al. (2019). Briefly, each purified bacterial isolate was then grown in Tryptic Soy Broth (TSB). Frozen stock of each isolate was prepared with TSB containing 15% glycerol as a cryoprotectant and maintained at −80°C. A total of 39 purified Gram-negative bacterial isolates were used as potential hosts for phage isolation.

For this study, fresh cultures were prepared in a 96-well microplate (Microplate I). Each well contained 200 μl of TSB and was inoculated with one bacterial isolate. After overnight incubation at 37°C, 50 μl of each culture in Microplate I was transferred into a new microplate (Microplate II) where each well contained 150 μl of TSB and 50 μl of the pH-adjusted filtered cover brine (a potential phage source). After the incubation at 37°C for 24 h, Microplate II was centrifuged (SH-3000 rotor, RC-5B centrifuge, Sorvall, Newtown, CT, United States) at 4,000 rpm, 4°C for 20 min. The supernatants from Microplate II were transferred into a new microplate (Microplate III). Spot tests were then performed by spotting 10 μl of supernatant from a well in Microplate III onto the corresponding bacterial lawn resulted from 100 μl overnight culture from the corresponding well in Microplate I. Primary phage-host relationships were indicated by positive spot-test plates after overnight incubation at 37°C. The resulting phage isolates underwent two rounds of plaque purification according to the method described by Lu et al. (2003a). The glycerol stocks of phages were prepared and stored at −80°C for later use.

### Identification of Phage Hosts

Distinct phage hosts were determined based on phage typing. Each host was then identified by partial *16S rRNA* gene sequencing and API 20E miniaturized biochemical testing as described by Pérez-Díaz et al. (2019). The *16S rRNA* amplicons were in average 700 bp in length. The substrate assimilations on the API20E strips were read after 24 and 48 h, and the interpretation of the results was done after 48 h. In the few cases were the *16S rRNA* sequencing analysis generated results different from the API20E analysis, a third identification tool was applied. The sequencing of the *dnaJ* housekeeping gene, applied as described by Pham et al. (2007), was utilized as a third identification tool. The *dnaJ* was sequenced for host no. 112, 113, 116, 222, and 231.

### Phage Characterization

Eight phage isolates along with their corresponding hosts were selected and used in a cross-infection experiment (Table 2) with spot tests to determine their host ranges. Based on host ranges, distinct phages were identified. Those phages were further characterized based on their morphology, major structural protein profiles, and restriction endonuclease digestion patterns using the methods previously described by Lu et al. (2003a) with minor modifications. Briefly, phage lysates were centrifuged at 4,000 × *g* for 20 min and filtered (0.45 μm pore size). The filtrates were treated with DNase I and RNase A. Phage particles were concentrated by polyethylene glycol precipitation and then purified by cesium chloride density gradient ultracentrifugation at 600,000 *g* for 6 h at 15°C. The ultracentrifuge-purified phages were used for electron microscopy analysis, sodium dodecyl sulfate-polyacrylamide gel electrophoresis (SDS-PAGE), and DNA extraction. Phage samples were negatively stained with 2% (w/v) aqueous uranyl acetate and examined by transmission electron microscopy at an accelerating voltage of 80 kV in the Center for Electron Microscopy at North Carolina State University. SDS-PAGE was carried out with boiled phage samples loaded onto NuPAGE precast gradient minigels (4-12% Bis-Tris, Invitrogen Corporation, Carlsbad, CA, United States). Phage DNA was prepared from the concentrated lysate using the phenol-chloroform extraction method, and digested with several restriction endonucleases (*EcoR*I, *EcoR*V, *Hind*III, *Msp*I, and *Swa*I) according to the supplier’s recommendations (New England BioLabs, Woburn, MA, United States). The resulting DNA fragments were separated on the 1% agarose gel containing 0.001% SYBR Safe DNA gel stain (Invitrogen) by gel electrophoresis in tris-borate-EDTA buffer at 70V (constant voltage) for 2.5 h.

### Phage Infection in Cucumber Juice

Cucumber juice was used as a model system to evaluate the infectivity of a phage active against *E. cancerogenus*. The cucumber juice was prepared from fresh cucumbers (size 3A) as described by Zhai et al. (under review) and filtered (0.45 μm pore size). Phage Φ107 was added to a tube containing cucumber juice and host *E*. *cancerogenus* 107 (10^5^ CFU/ml) at the initial multiplicity of infection (MOI) 1 or 100. Cucumber juice containing only host (without phage) was used as a control. After briefly mixed, each tube was incubated in water bath at 37°C. Hourly samples were taken from each tube and then properly diluted before plated onto tryptic soy agar plates. After incubated at 37°C overnight, each plate was examined to obtain plate counts used for calculation of cell concentration.

### Statistical Analysis

One-way Analysis of variance (ANOVA) was performed using Statistica for Windows (StatSoft, Tulsa, OK, United States). Tukey’s HSD test was used to compare the mean values of data for significant difference (*P* < 0.05).

## Results

### Bacterial Concentrations During the Fermentation

**FIGURE 1:**
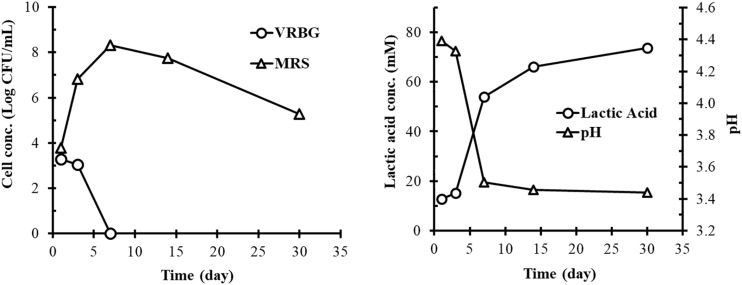
Bacterial concentrations **(left)** and lactic acid production and pH **(right)** in the commercial cucumber fermentation studied. Plate counts from VRBG (∘) and MRS (Δ) agar plates represent the concentrations of Gram-negative bacteria and lactic acid bacteria, respectively. Lactic acid production (∘) and pH change (Δ) are shown in the right panel. Data points represent the mean and standard deviations of samples collected from the fermentation tank.

The concentrations of Gram-negative bacteria and LAB on fresh cucumbers were 5.4 and 4.2 Log CFU/g, respectively (data not shown). Figure 1 shows the concentration profiles of bacteria during the fermentation over a 30-day period. Gram-negative bacterial concentration was 3.3 and 3.0 Log CFU/ml on days 1 and 3 of the fermentation, respectively. Thereafter, the cell concentration rapidly decreased below detectable levels by day 7. In contrast, LAB concentration was 3.8 Log CFU/ml on day 1, rapidly increased to 6.8 Log CFU/ml on day 3, reached the maximum (8.3 Log CFU/ml) on day 7, and then gradually decreased to 5.5 Log/ml on day 30. It was noticed that LAB concentration was slightly higher than Gram-negative bacterial concentration on day 1. The overall bacterial profile is typical of a commercial cucumber fermentation (Pérez-Díaz et al., 2016).

### Chemical Analyses

The fermentation started at pH 4.4 in the recycled cover brine. Rapid lactic acid production was observed between day 3 and day 7, which resulted in pH decrease to 3.5 (Figure 1). These changes were attributed to the rapid growth of LAB and resulting acid production during this period of time (Figure 1). After day 7, lactic acid concentration continued to increase (but not as rapidly as earlier) and pH decreased slightly. On day 30, lactic acid concentration and the resulting pH reached 74 mM and 3.4, respectively, which were typical of cucumber fermentations.

### Isolation of Gram-Negative Bacteria and Their Phages

A total of 39 Gram-negative bacterial isolates were obtained from VRBG plates. Nineteen (nearly 50%) of them were obtained from day 1, and 20 from day 3. No colonies were observed on VRBG plates on day 7 or thereafter, indicating that Gram-negative bacteria died off or their concentration was below the detection limit on day 7. This could be due to inhibitory effect of lactic acid produced, the resulting low pH (3.5), and possible other factors such as phage infection as discussed below. Using the 39 Gram-negative bacterial isolates as potential hosts and the cover brine samples as potential phage source, 26 phage isolates were obtained by spot tests (Table 1). The first 11 phages listed in Table 1 were obtained on day 1, and the remaining 15 phages were obtained on day 3. Since no Gram-negative bacteria were isolated on day 7 and thereafter, no effort was made to isolate phages against Gram-negative bacteria on and after day 7.

**TABLE 1:** Phages and their corresponding hosts.

Phage	Host						
ID	ID	ID by *16S rRNA* sequencing	Accession no.	% Identity/coverage	Identification by API 20E	% Identity	Comments
Φ102	102	*Citrobacter freundii*	MH045681	100/99	*Citrobacter freundii*	84.2	Very good identification to the genus
Φ103	103	*Enterobacter cloacae*	MH045682	100/100	*Enterobacter cloacae*	97.7	Good identification
Φ107	107	*Enterobacter* sp.	MH045685	99/100	*Enterobacter cancerogenus*	99.3	Very good identification
Φ109	109	*Pantoea* sp.	MH045687	100/99	*Pantoea* sp.	91.1	Good Identification
Φ111	111	*Providencia rettgeri*	MK514946	100/100	*Providencia rettgeri*	99.9	Good identification
Φ112	112	*Enterobacter asburiae*	MH045689	87/90	*Serratia ficaria*	59.8	Invalid identification
Φ113	113	*Pseudocitrobacter anthropi*	MK514947	100/100	*Pseudomonas fluorescens/putida*	75	Good identification to the genus
Φ115	115	*Enterobacter cloacae* subsp. *cloacae*	MH045692	100/98	*Enterobacter cloacae*	94	Good identification
Φ116	116	*Enterobacter* sp.	MK514954	99.88/100	*Leclercia adecarboxylata*	85.6	Low discrimination
Φ117	117	*Providencia rettgeri*	MK514948	100/100	*Providencia rettgeri*	99.2	Very good identification
Φ119	119	*Enterobacter cloacae* subsp. *cloacae*	MH045695	100/99	*Enterobacter cloacae*	97.7	Good identification
Φ220	220	*Enterobacteriaceae*	MH045696	100/97	*Escherichia vulneris*	80.5	Acceptable identification
Φ221	221	*Enterobacter cloacae* subsp. *cloacae*	MH045697	100/98	*Enterobacter cloacae*	97.7	Good identification
Φ222	222	*Enterobacter* sp.	MH045698	94/100	*Leclercia adecarboxylata*	85.6	Low discrimination
Φ225	225	*Enterobacter cloacae* subsp. *cloacae*	MH045701	100/97	*Enterobacter cloacae*	97.7	Good identification
Φ226	226	*Leclercia adecarboxylata*	MH045702	100/97	*Leclercia adecarboxylata*	85.6	Low discrimination
Φ227	227	*Citrobacter freundii*	MH045703	100/98	*Citrobacter freundii*	99.9	Excellent identification
Φ228	228	*Enterobacter cloacae* subsp. c*loacae*	MK514949	100/100	*Enterobacter cloacae*	97.7	Good identification
Φ230	230	*Enterobacter cloacae* subsp. *cloacae*	MK514950	100/99	*Enterobacter cloacae*	97.7	Good identification
Φ231	231	*Enterobacter* sp.	MH045705	98/99	*Leclercia adecarboxylata*	85.6	Low discrimination
Φ232	232	*Citrobacter freundii*	MK514951	89/99	*Citrobacter freundii*	99	Excellent identification
Φ233	233	*Enterobacteriaceae*	MH045706	99/96	*Escherichia vulneris*	99.1	Very good identification
Φ235	235	*Enterobacter* sp.	MH045708	100/98	*Enterobacter cloacae*	97.7	Good identification
Φ237	237	*Enterobacter cloacae* subsp. *cloacae*	MH045710	100/97	*Enterobacter cloacae*	97.7	Good identification
Φ238	238	*Enterobacter* sp.	MH045711	100/97	*Enterobacter cloacae*	97.7	Good identification
Φ239	239	*Leclercia adecarboxylata*	MH045712	100/97	*Leclercia adecarboxylata*	85.6	Low discrimination

*All isolates were obtained from a commercial cucumber fermentation in North Carolina, United States.*

**TABLE 2:** Host ranges of 8 enterobacterial phages.

ID	Host identification^a^	Phage identification							
		Φ107	Φ115	Φ220	Φ225	Φ226	Φ231	Φ238	Φ239
107	*Enterobacter cancerogenus*	+	+		+	+		+	
115	*Enterobacter cloacae* subsp. *cloacae*	+	+		+				
220	*Enterobacteriaceae*			+					
225	*Enterobacter cloacae* subsp. *cloacae*				+				
226	*Leclercia adecarboxylata*					+			
231	*Enterobacter* sp.	+					+		
238	*Enterobacter cloacae*							+	
239	*Leclercia adecarboxylata*								+

**^*a*^Host identification shown in this table is based on 16S rRNA gene sequencing*.*

### Host Identification

Among the 39 Gram-negative bacterial isolates, 26 (or 67%) of them were found to be sensitive to phage attack (Table 1). Based on the *16S rRNA* gene sequence analysis, 23 (or 88%) of the hosts belong to the *Enterobacteriaceae* family, including 15 (or 65%) *Enterobacter* spp. Among the 23 hosts, 8 were identified as *Enterobacter cloacae*, 1 as *Enterobacter asburiae*, 6 as *Enterobacter* sp., 3 as *Citrobacter freundii*, 2 as *Leclercia adecarboxylata*, 1 as *Pantoea* sp., 1 as *Escherichia vulneris*, and one (ID 220) as another member in *Enterobacteriaceae* family identified as *Escherichia vulneris* by the API 20E system (Table 1).

The host identification using the API 20E system is in general agreement with that by the *16S rRNA* gene sequencing (Table 1). That is, 23 host isolates belong to *Enterobacteriaceae*, one to *Pseudomonas*, and two to *Providencia*. Among the 26 hosts, 20 were identified to the species level, yielding excellent, very good, good, and acceptable species identifications in 2, 4, 13, and 1 case(s), respectively (Table 1). Five hosts (ID 116, 222, 226, 231, and 239) were identified as *L. adecarboxylata* with low discrimination (85.6% identity). But two of them (ID 226 and 239) were also identified as *L. adecarboxylata* by the *16S rRNA* gene sequence analysis, and another three (ID 116, 222, and 231) as *Enterobacter cloacae*. Hosts 116, 222, and 231 were also identified as *Enterobacter* sp. by *dnaJ* sequencing with 89% query identity over 88–96% of the homolog sequences. The *dnaJ* sequences obtained for hosts 116, 222, and 231 can be located in GenBank using accession numbers MG678924, MG678933, and MG678927, respectively. It was noticed that the host (ID 112) with invalid identification (*Serratia ficaria*) was identified as *E. asburiae* by *16S rRNA* gene and the *dnaJ* sequence analyses. The accession number for the *dnaJ* sequence obtained from host 112 can be located with accession number MG678921 in GenBank.

### Phage Characterization

**FIGURE 2:**
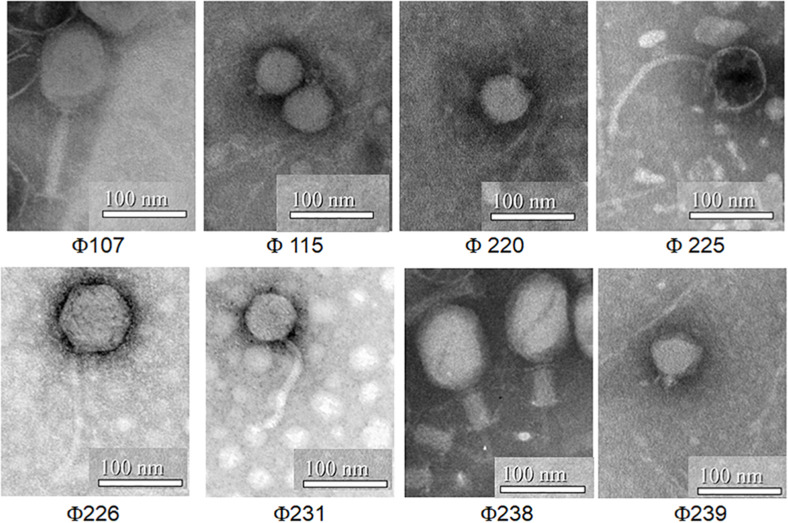
Transmission electron micrographs of eight phages infecting Gram-negative bacteria in a commercial cucumber fermentation.

Among the 26 phage isolates infecting Gram-negative bacteria, eight phages with unique host range were selected for characterization. The electron micrographs (Figure 2) show that all eight phages are tailed phages with icosahedral heads in the viral order *Caudovirales*. Two phages (Φ107, and Φ238) belong to the *Myoviridae* family, three phages (Φ231, Φ225, and Φ226) belong to the *Siphoviridae* family, and other three phages (Φ115, Φ239, and Φ220) belong to the *Podoviridae* family. Table 2 showed that the eight phages have different host ranges. The host identification was based on *16S rRNA* gene sequencing. Unfortunately, *16S rRNA* gene sequencing only identified the family name (*Enterobacteriaceae*) for host 220.

**FIGURE 3:**
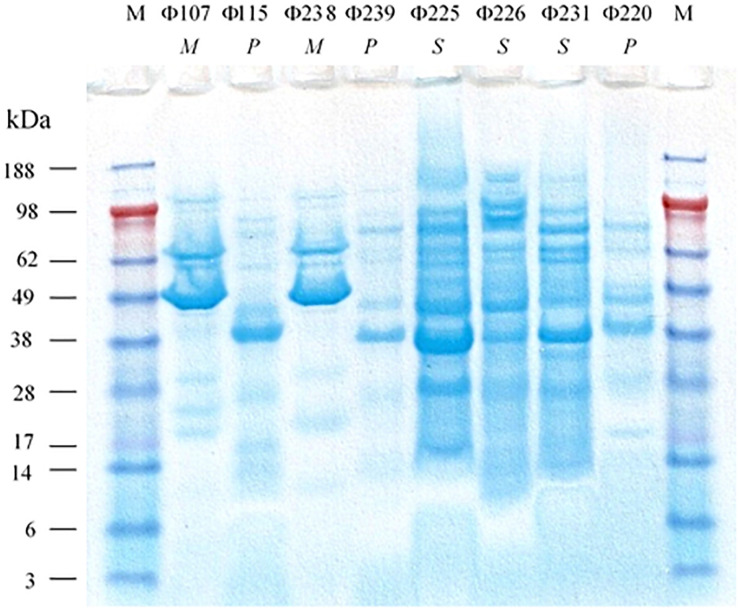
SDS-PAGE analysis of structural proteins of eight phages isolated from a commercial cucumber fermentation. Lane M, molecular mass standard; Lanes 1 to 8: Φ107, Φ115, Φ238, Φ239, Φ225, Φ226, Φ231, and Φ220. *M*, *P*, and *S* stand for *Myoviridae*, *Podoviridae*, and *Siphoviridae*, respectively.

**FIGURE 4:**
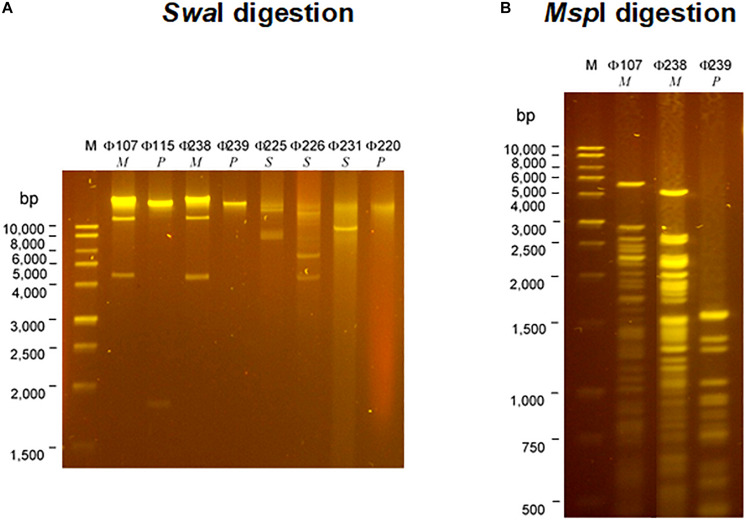
Restriction analysis of DNAs of eight selected phages isolated from a commercial cucumber fermentation. Phage DNAs were digested with *Swa*I **(A)** and *Msp*I **(B)**. Lane M, 1-kb DNA ladder; Lanes 1–8: Φ107, Φ115, Φ238, Φ239, Φ225, Φ226, Φ231, and Φ220; Lanes 9–11: Lanes 1–8: Φ107, Φ238, and Φ239.

The SDS-PAGE analysis (Figure 3) showed that the structural protein profiles of the eight phages. Interestingly, the structural protein banding patterns of the two *Myoviridae* phages (Φ107, and Φ238) are very similar (but not identical) to each other. The banding patterns of the three *Siphoviridae* phages (Φ225, Φ226, and Φ231) are also similar to one another, but different from those of *Myoviridae* or *Podoviridae* phages which have fewer protein bands. The banding patterns of three *Podoviridae* phages (Φ115, Φ239, and Φ220) are only slightly different from one another, but very different from the other five phages. The analysis of restriction fragment length polymorphism (RFLP) was carried out using two restriction endonucleases, *Swa*I and *Msp*I. The two *Myoviridae* phages (Φ107, and Φ238) showed the same restriction banding pattern from *Swa*I digestions (Figure 4A), but very different banding patterns from *Msp*I digestions (Figure 4B). The three *Siphoviridae* phages (Φ225, Φ226, and Φ231) showed different restriction banding patterns from *Swa*I digestion (Figure 4A). Those banding patterns are not only different from one another, but also different from those of other phages. Digestion of the DNA from *Podoviridae* phage Φ115 with *Swa*I generated a fragment of 1900 bp (Figure 4A). The DNA from another *Podoviridae* phage Φ239 cannot be digested by *Swa*I (Figure 4A) but can be digested by *Msp*I generating more than 10 restriction fragments (Figure 4B). In contrast, the DNA of *Podoviridae* phage Φ220 could neither be digested by *Swa*I (Figure 4A) nor by *Msp*I (data not shown).

### Phage Infection in Cucumber Juice

**FIGURE 5:**
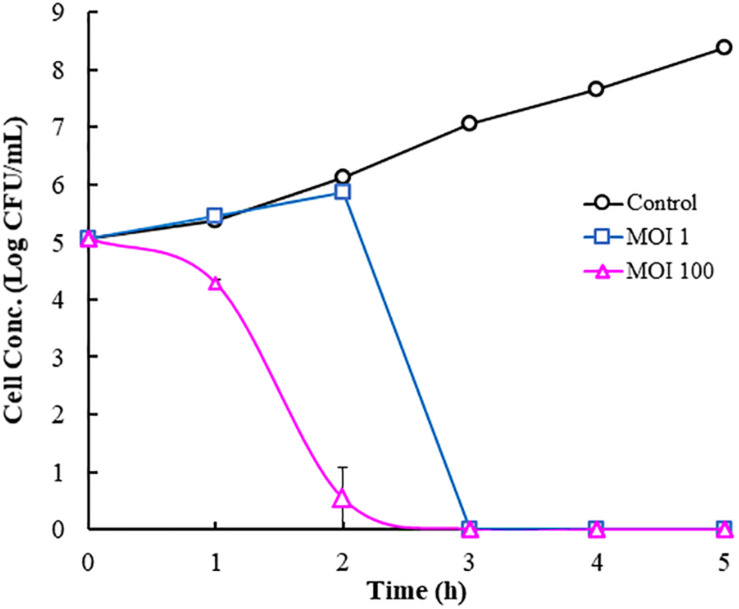
Phage infection by Φ107 against *Enterobacter cancerogenus* 107 in cucumber juice.

Cucumber juice contained most of the nutrients found in the whole cucumber and thus represents whole cucumbers. Because cucumber juice is a liquid and easy to work with, it was used as a model system to evaluate the infectivity of phage Φ107, which is active against its host *E. cancerogenus* 107. No salt, acid, and/or LAB were added to the model system to exclude their inhibitory effects against Gram-negative bacteria. During the first 2 h, the host concentrations in the control tube (without phage) increased from 10^5^ to 1.4 × 10^6^ CFU/ml (Figure 5). While the cell concentration in the infection tube at MOI 1 was similar to that of the control, the cell concentration in the infection tube at MOI 100 decreased rapidly and resulted in more than 4-log reduction (Figure 5). During the third hour, the cell concentration in the control tube continued to increase and reached 10^7^ CFU/ml. In contrast, the cell concentrations in both infection tubes, regardless of the initial MOI, decreased to the undetectable level (below 10 CFU/ml). That is, phage infection (regardless of the initial MOI) caused rapid cell death within 3 h and resulted in 5-log reductions in cell concentration compared to the initial cell concentration. Different from cells in the infection tubes, cells in the control tube continued to increase over next 2 h and reached 2.4 × 10^8^ CFU/ml.

## Discussion

The bacterial concentrations on fresh cucumbers and the fermentation profile obtained from the tank studied are in line with a typical industrial cucumber fermentation (Pérez-Díaz et al., 2015). The plate counts for presumptive LAB from MRS agar plates corresponding to the samples collected on day 1 of the fermentation are artificially higher due to the fact that *Exiguobacterium*, *Staphylococcus*, *Bacillus*, *Clostridium*, and *Aerococcus* can also proliferate in such medium (Pérez-Díaz et al., 2019). The plate counts on VRBG and MRS plates from day 1 samples are likely impacted by the bacterial counts on the fresh cucumbers and inside the cucumbers due to the possible internalization from the skin to the endocarp during the equilibration between cucumbers and the cover brine. The observation that LAB concentration was higher than Gram-negative bacterial concentration on day 1 is because the recycled cover brine from previous cucumber fermentations already contained some LAB.

An abundance of phages against *Enterobacteriaceae* was found in the industrial fermentation cover brines, particularly those capable of infecting *Enterobacter* (Table 1). Phages were isolated from cover brines collected on days 1 and 3, when the pH was above 4.3, thus it is unknown if such phages can survive later in fermentations as the pH declines to 3.3 and their hosts died off. However, the prevalence of *Enterobacteriaceae* in the early stage of cucumber fermentations has been documented in the literature (Pederson and Albury, 1950; Mundt et al., 1967; Mundt and Hammer, 1968; Mundt, 1970; Pérez-Díaz et al., 2019). It is well known that *Enterobacteriaceae* produce gas when fermenting glucose and fructose (the major sugars in cucumbers), which can lead to cucumber bloater defect (Zhai et al., under review). The critical period for susceptibility of a fermentation to bloater defect is from day 1 to day 32 (Fleming et al., 1978).

In line with the prevalence of *Enterobacteriaceae* is the finding that at least 58% of phage hosts were *Enterobacter* species. A recent study on nine commercial cucumber fermentations showed that *Enterobacter* species were highly prevalent in the fermentations on days 1 and 3 (Pérez-Díaz et al., 2019). Among the *Enterobacter* isolates, 8 were identified as *E. cloacae*. Several studies reported that *E. cloacae* is the most frequently isolated *Enterobacteriaceae* from spoiled cucumber fermentations (Veldhuis and Etchells, 1939; Etchells, 1941; Pérez-Díaz et al., 2019). This Gram-negative facultative anaerobe is able to produce carbon dioxide and hydrogen in spoiled fermentations with an increased incidence of bloater defect (Etchells, 1941). *E. cloacae* is also able to convert lactic acid to propionic and butyric acids leading to an increase in pH (Samish et al., 1957, 1963; Franco et al., 2012; Breidt et al., 2013b; Franco and Pérez-Díaz, 2013; Pérez-Díaz et al., 2019).

Some isolated phage hosts can also induce spoilage or be a cause for a food safety concern. *Pseudomonas* species produce protease and lipase that can cause spoilage in meat, milk and dairy products (Walker, 1988). *Citrobacter freundii*, *E. cloacae*, *E. vulneris*, *Pantoea agglomerans*, and *Providencia rettgeri* are opportunistic human pathogens. *Pseudomonas fragi* and *E. cancerogenus* are true bacterial pathogens. It is speculated that if the Gram-negative bacteria described above survive in a cucumber fermentation and rapidly grow, the activity of LAB may be impaired. Further research is needed to test this hypothesis. If the presence of Gram-negative bacteria significantly impairs LAB activity, there will be more reasons to eliminate and/or control Gram-negative bacteria in the early stage of the fermentation.

Our observation that Gram-negative bacteria were excluded early in the fermentation is in agreement with those reported in the literature (Pérez-Díaz et al., 2019). The disappearance of Gram-negative bacteria in cucumber fermentation as well as other vegetable fermentations are often attributed only to chemical factors such as high salt concentration, the acids produced by LAB, and the resulting low pH in the fermentation (Samish et al., 1963; Garrido-Fernández et al., 1997; Pérez-Díaz et al., 2019). However, other factors may also play a role in the bacterial mortality. Our data showed that 67% of the Gram-negative bacterial isolates were infected by diverse phages. Such high percentage of infected bacterial isolates is unexpected. We previously studied phages infecting LAB in the same cucumber fermentation and found that the percentage of LAB infected by phages was much lower (Lu et al., 2012). On day 1 and day 3, 0 and 13% of LAB isolates were attacked by phages, respectively. Even on day 14 (when the highest LAB phage activity was observed), only 28% of LAB isolates were attacked by phages (Lu et al., 2012). Thus, the mortality of the Gram-negative bacteria caused by their phages cannot be overlooked. It is possible that phages played a significant role in the death of Gram-negative bacteria in the early stage of the fermentation, thereby facilitating the dominance of LAB and reducing bloater incidence.

The eight characterized phages showed distinct host ranges, indicating that they are different from each other. It is noticed that 4 phages Φ107, Φ115, Φ225, and Φ238 infected at least one *E. cloacae* strain. Specifically, Φ225 infected two *E*. *cloacae* strains while Φ107, Φ115, and Φ238 infected one of *E. cloacae* strains. In contrast, Φ107, Φ115, and Φ238 are capable of infecting two different species, *E. cloacae* and *E. cancerogenus*. Interestingly, Φ226 is capable of infecting bacteria in two different genera (*Enterobacter* and *Leclercia*). In the past, most phages were found species-specific and rarely crossing species boundaries (Beumer and Robinson, 2005). However, recent studies showed that some phages have a broad host range, crossing species or even genus barrier. We previously reported that many LAB phages isolated from vegetable fermentations are capable of infecting across *Lactobacillus* species, and two phages are capable of infecting *Weissella cibaria*, *Lactobacillus plantarum*, and *Lactobacillus brevis* (Lu et al., 2003b, 2012). Several other researchers showed that certain phages such as SFP10 and GG32 are capable of infecting both *Salmonella enterica* and *E. coli* O157:H7 (Park et al., 2012; Chae et al., 2016). Phages that are capable of crossing genera may use receptors, intermediary functions, or both, common to a wide range of bacteria (Bielke et al., 2007). Broad-host-range phages may play a key role in horizontal gene transfer between different species or genera of bacteria, thereby promoting genetic diversity in microbial communities (Jensen et al., 1998). Beumer and Robinson (2005) demonstrated that a broad-host-range, generalized transducing phage can acquire and carry *16S rRNA* gene sequences from bacteria belonging to different genera (Beumer and Robinson, 2005). While different phages can be differentiated by their host ranges, different bacterial species or strains can be distinguished by phage typing. Hosts 115, 225, and 238 are all identified as *Enterrobacter cloacae* or *Enterobacter cloacae* subsp. *cloacae* (Table 2). They differ in their susceptibilities to different sets of phages. Host 115 is sensitive to three different phages (Φ107, Φ115, and Φ225) while hosts 225 and 238 are sensitive to only one phage (Φ225 and Φ238, respectively). Therefore, these three hosts are different strains of *E. cloacae*. Hosts 226 and 239 are both identified as *L*. *adecarboxylata*, but they are sensitive to different phages, and thus they are different strains. Based on phage typing, the eight phage hosts are different from each other although they all belong to *Enterobacteriaceae* family. An interesting finding from SDS-PAGE analysis is that phages from the same family have similar structural protein profiles while phages from different families have very different structural protein profiles. We have not seen such interesting features when we studied LAB phages isolated from the same fermentation. There, LAB phages from the same family did not show similar structural protein profiles (Lu et al., 2012). Even though in this study phages from the same family share similar protein profiles, their protein profiles are not identical. Thus, the eight phages isolated in this study are different phages. Additionally, the RFLP results showed that the eight phages are genetically distinct, which provided a glimpse of the genetic diversity of phages against Gram-negative bacteria in cucumber fermentations. Further research will be needed to reveal the extent of the genetic diversity of phages from cucumber fermentations and to study the life cycles of these phages.

Because *Enterobacter* including *E. cloacae* and *E*. *cancerogenus* was highly prevalent in cucumber fermentations (Pérez-Díaz et al., 2019), *Enterobacter* is an important group of the gas-producing Gram-negative bacteria contributing to cucumber bloating (Veldhuis and Etchells, 1939; Etchells, 1941; Samish et al., 1957; Samish et al., 1963; Zhai et al., under review). In this study, 15 (or 58%) of the isolated phages infected *Enterobacter*. It would be interesting to evaluate the effectiveness of one of those phages on the elimination of its host in cucumber juice as a model system. Host 107 (*E. cancerogenus*) was identified as one of the culprits in the causation of bloater defect in cucumber fermentations. It was able to produce high concentration of carbon dioxide in cucumber juice medium (Zhai et al., under review). In this study, Φ107 was selected to evaluate its effectiveness on the elimination of host 107 in cucumber juice used as a model system. The results showed that the infection of *E. cancerogenus* 107 by its phage Φ107 at the MOI of 1 or 100 effectively eliminated the host in cucumber juice within a short period of time in the absence of LAB and high salt concentration, the two factors thought to be critical in the die off of Gram-negative bacteria in cucumber fermentations. Our data clearly demonstrates that phage infection could potentially eliminate the target bacterial hosts effectively in cucumber fermentations. Since a diverse group of Gram-negative bacteria were found in the cucumber fermentation, a phage cocktail against those bacteria needs to be tested in the model system and applied in cucumber fermentations in order to completely prevent bloater formation. It is concluded that the abundant and varied phages active against *Enterobacter* and many other Gram-negative bacteria in cucumber fermentations can contribute to the eradication of those bacteria and induce changes in the microbial community, thereby promoting the dominance of LAB and also potentially minimizing bloater defect during cucumber fermentations. Therefore, the role of phages in the perishing of Gram-negative bacteria in cucumber fermentations cannot be overlooked and should be further investigated. It is likely that a combination of both chemical and biological factors including phages lead to the disappearance of Gram-negative bacteria and the dominance of LAB in cucumber fermentations.

## Conclusion

The data from this study showed that abundant and diverse phages were present in a commercial cucumber fermentation, and 67% of Gram-negative bacterial isolates were sensitive to phage infection. These results suggest that phage infection could cause substantial mortality in the indigenous Gram-negative bacterial population in cucumber fermentations, and thus potentially influence the bacterial ecology, minimize bloater defect, and promote the quick dominance of LAB in cucumber fermentations. This study provides new insights into the functional role of phages in the dynamic process of cucumber fermentations. More studies are needed to further explore the phage ecology in other commercial cucumber fermentations in the same and different geographic locations, and to understand the factors shaping the microbial ecology of vegetable fermentations. To our knowledge, this is the first study to explore the ecology of phages infecting Gram-negative bacteria in commercial cucumber fermentations.

## Data Availability Statement

All datasets generated for this study are included in the article/supplementary material.

## Author Contributions

ZL isolated and characterized phages and wrote the manuscript. IP-D and JH isolated and identified phage hosts. FB assisted with the electron microscopy analysis. IP-D and FB were involved in the manuscript revision. All authors contributed to the article and approved the submitted version.

## Conflict of Interest

The authors declare that the research was conducted in the absence of any commercial or financial relationships that could be construed as a potential conflict of interest.
